# Warmer temperatures enhance beneficial mutation effects

**DOI:** 10.1111/jeb.13642

**Published:** 2020-06-23

**Authors:** Xiao‐Lin Chu, Da‐Yong Zhang, Angus Buckling, Quan‐Guo Zhang

**Affiliations:** ^1^ State Key Laboratory of Earth Surface Processes and Resource Ecology and MOE Key Laboratory for Biodiversity Science and Ecological Engineering College of Life Sciences Beijing Normal University Beijing China; ^2^ ESI & CEC Biosciences University of Exeter Penryn UK

**Keywords:** fitness, mutation accumulation, mutational effects, positive selection

## Abstract

Temperature determines the rates of all biochemical and biophysical processes, and is also believed to be a key driver of macroevolutionary patterns. It is suggested that physiological constraints at low temperatures may diminish the fitness advantages of otherwise beneficial mutations; by contrast, relatively high, benign, temperatures allow beneficial mutations to efficiently show their phenotypic effects. To experimentally test this “mutational effects” mechanism, we examined the fitness effects of mutations across a temperature gradient using bacterial genotypes from the early stage of a mutation accumulation experiment with *Escherichia coli*. While the incidence of beneficial mutations did not significantly change across environmental temperatures, the number of mutations that conferred strong beneficial fitness effects was greater at higher temperatures. The results therefore support the hypothesis that warmer temperatures increase the chance and magnitude of positive selection, with implications for explaining the geographic patterns in evolutionary rates and understanding contemporary evolution under global warming.

## Graphical abstract

Warmer temperatures could speed natural selection by increasing the amount of beneficial mutations (a) or enhancing the fitness effects of beneficial mutations (b). The second scenario was supported by the present study that examined the fitness effects of spontaneous mutations across a temperature gradient using *Escherichia coli* genotypes from a short‐term mutation accumulation experiment.

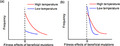

## 1 INTRODUCTION

**Figure 1:**
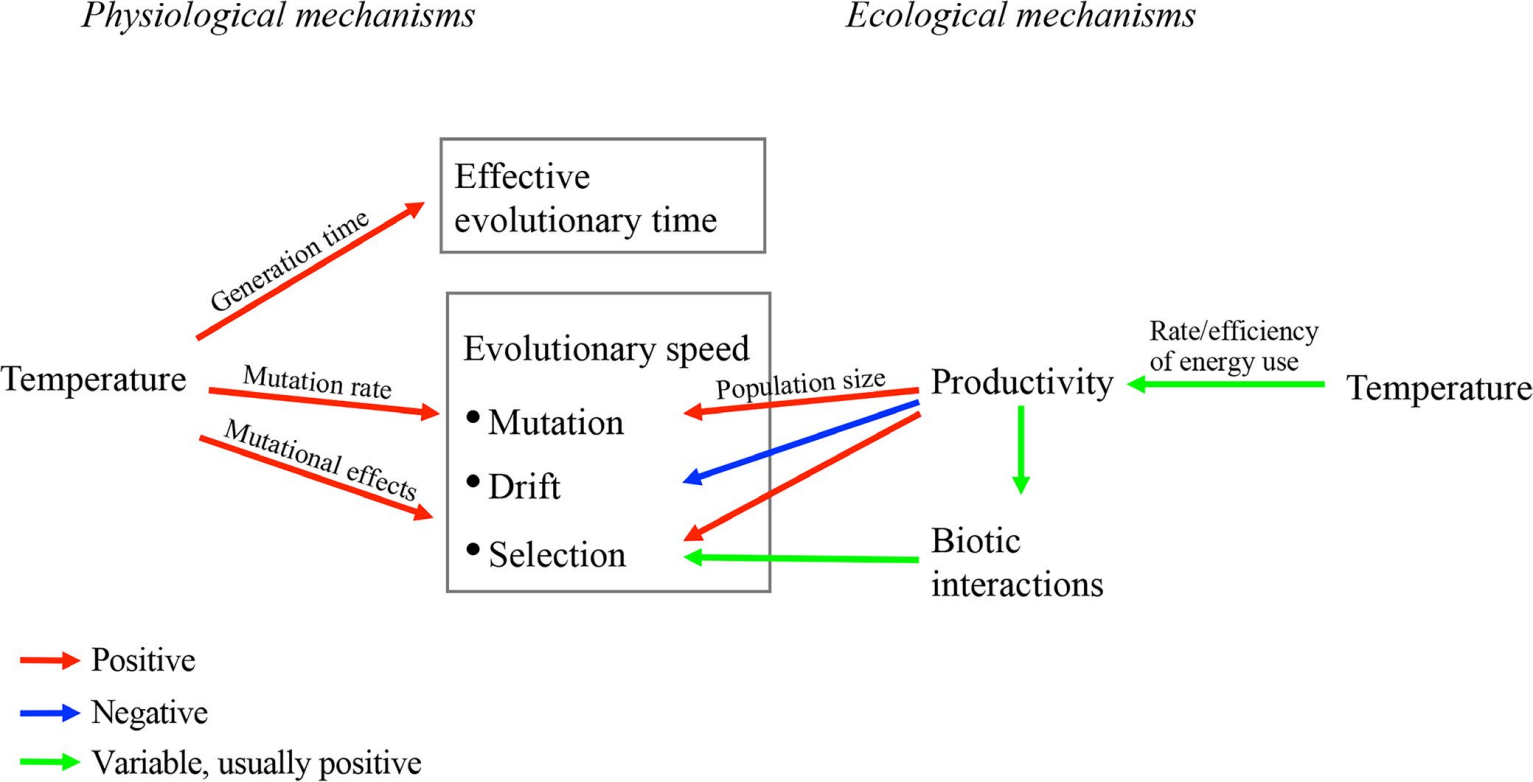
A summary of possible mechanisms through which temperature can affect evolutionary speed

Temperature is a crucial factor determining the rates of evolutionary processes that drive biodiversity patterns including the latitudinal diversity gradient (Allen & Gillooly, 2006; Allen, Gillooly, & Brown, 2007; Brown, 2013; Clarke & Gaston, 2006; Connell & Orias, 1964; Dobzhansky, 1950; Fischer, 1960; Gaston, 2000; Jablonski, Roy, & Valentine, 2006; McKenna & Farrell, 2006; Pianka, 1966; Rohde, 1992; Stehli, Douglas, & Newell, 1969). Mechanisms underlying the temperature effects on evolutionary speed fall into two categories (Figure 1). The ecological effects, involving indirect consequences of temperature, are mediated by changes in habitat productivity and the strength or complexity of biotic interactions. Higher temperatures often, though not always, increase ecosystem productivity (Allen et al., 2007; Fischer, 1960; Gaston, 2000; Rohde, 1992), which would lead to larger population sizes and thus an increase of mutational supply and a decrease of the importance of drift relative to selection (Gillespie, 1998). Moreover, greater productivity usually leads to more intense and complex biotic interactions, both within and between species (Abrams, 1995; Harpole & Tilman, 2007; Huston, 1979; Rosenzweig, 1995), and the stronger biotic interactions may often create more opportunities for fluctuating selection, resulting in faster evolution (Bell, 2008; Brown, 2013; Connell & Orias, 1964; Rohde, 1992; Thompson, 2005; Van Valen, 1973). Those ecological effects may often, but not always, lead to a positive temperature–evolutionary speed relationship (Connell & Orias, 1964; Dobzhansky, 1950; Dowle, Morgan‐Richards, & Trewick, 2013; Fischer, 1960; Pianka, 1966; Schemske, Mittelbach, Cornell, Sobel, & Roy, 2009; Vazquez & Stevens, 2004).

Meanwhile, the more direct, physiological, consequences of increased temperatures may have consistently positive effects on the rate of evolution (Figure 1). For example, higher temperatures (within the normal ranges for organisms) can shorten generation times (Gillooly, Charnov, West, Savage, & Brown, 2002; Martin & Palumbi, 1993; Rohde, 1992) and elevate mutation rates (Chu et al., 2018; Gillooly, Allen, West, & Brown, 2005; Martin & Palumbi, 1993; Ryan & Kiritani, 1959; Zuckerkandl & Pauling, 1965). It has also been argued that warmer temperatures may speed natural selection, most likely by affecting the fitness effects of mutations (Fischer, 1960). However, this “mutational effects” hypothesis has been largely overlooked; and empirical evidence is lacking.

Fischer (1960) proposed that the fact warmer environments allowing a larger range of physiological and structural variants to survive may lead to faster natural selection. In other words, Fischer envisioned a scenario that lower temperatures render the otherwise beneficial mutations to become deleterious, reducing the availability of beneficial mutations. We may also imagine another scenario that lower temperatures simply reduce the size of fitness effects of beneficial mutations. The temperature influences on mutational effects may arise naturally because of the ubiquitous effects of temperature on biochemical and biophysical processes that life activities depend on, for example enzyme reaction, protein synthesis and ligand‐binding processes (DePristo, Weinreich, & Hartl, 2005; Echave & Wilke, 2017; Hochachka & Somero, 2002; Malerba & Marshall, 2019; Padfield, Yvon‐Durocher, Buckling, Jennings, & Yvon‐Durocher, 2016; Schaum, Buckling, Smirnoff, Studholme, & Yvon‐Durocher, 2018; Vacca et al., 2004). Low temperatures could lead to strong constraints on many, if not all, physiological functions. Therefore, a mutation that improves a specific biological function may likely fail to improve the overall growth performance due to the limitation of other functions, or even reduce organism growth if it incurs certain fitness costs. By contrast, the fitness of an organism at higher, relatively benign, temperatures may be limited by a smaller number of physiological constraints; hence, the potentially beneficial mutations would have greater chances to actually confer the fitness benefits. Note that very high temperatures that are stressful for organisms may instead allow a smaller range of mutations to survive and decrease the likelihood of beneficial mutations, where protein stability but not the rates of physiological processes becomes the major determinant of organism growth performance (Berger, Stangberg, & Walters, 2018; Chen & Shakhnovich, 2010; Dandage et al., 2018).

The present study experimentally tests the hypothesis that warmer temperatures enhance beneficial mutation effects. This question has been poorly understood, while previous research on the temperature dependence of mutational effects usually focused on deleterious mutations and the influences of stressful thermal conditions (Baer et al., 2006; Bank, Hietpas, Wong, Bolon, & Jensen, 2014; Berger et al., 2018; Dandage et al., 2018; Goho & Bell, 2000; Trindade, Sousa, & Gordo, 2012). Positive selection which drives long‐term adaptive evolution depends on the occurrence of beneficial mutations, and natural populations are typically located in benignly hot and modestly cold environments (Brown, 2013; Fischer, 1960; Rohde, 1992). Therefore, a better understanding of how normal range temperatures affect beneficial mutation effects would be crucial.

## 2 MATERIALS AND METHODS

### 2.1 Mutation accumulation

Mutation accumulation (MA) experiments have long been used for studying the fitness consequences of spontaneous mutations. MA experiments with bacteria involve propagating clonal populations through repeated single‐individual bottlenecks, during which the effective population size is extremely low, and thus, selection is weak relative to drift. It is expected that all mutations present in a population, except lethal ones, may reach fixation in a nearly neutral fashion (Baer et al., 2006; Eyre‐Walker & Keightley, 2007; Halligan & Keightley, 2009; Kibota & Lynch, 1996; Kondrashov & Houle, 1994; Morgan, Ness, Keightley, & Colegrave, 2014; Shewaramani et al., 2017; Szafraniec, Borts, & Korona, 2001). While the fitness effects of total mutations would be deleterious in the long run as most spontaneous mutations would be detrimental, short‐term experiments may obtain MA lines with fitness gains, reflecting the occurrence of beneficial mutations (Dickinson, 2008; Trindade, Perfeito, & Gordo, 2010).

Our MA experiment was conducted with the bacterial strain *Escherichia coli* B REL606 *mutS,* which is a mutator derivative of the wild‐type strain. This strain was constructed by P1 transduction of a disrupted allele of *mutS*, *mutS*::Tn5, into REL606 (Siegel, Wain, Meltzer, Binion, & Steinberg, 1982). The *mutS* protein is involved in the mismatch repair system by recognizing and binding to mispaired nucleotides. A total of 60 MA lines went through 30 bottlenecks at three temperatures, 25, 28 and 37°C, with 20 replicates at each temperature (Chu et al., 2018). In the present study, bacterial genotypes from bottleneck 10 of all the 60 MA lines were investigated. Here, we did not use genotypes from longer periods of MA because we were concerned that fitness effects of any beneficial mutations would be masked by the increasing numbers of accumulated deleterious mutations (Long, Paixão, Azevedo, & Zufall, 2013; Trindade et al., 2010; Vassilieva, Hook, & Lynch, 2000). Based on previous MA experiments (Dickinson, 2008; Trindade et al., 2010), we expected that, with a total of 60 bacterial genotypes from the very early stage of our MA experiment, more than 10 genotypes may show fitness gain relative to the ancestral strain.

### 2.2 Fitness assays

The fitness of each of the 60 MA line and the ancestor, relative to a reference bacterial strain (an Ara + revertant from the ancestral strain), was measured via head‐to‐head competition assays (Lenski, Rose, Simpson, & Tadler, 1991). The assays were carried out across six temperatures, 21, 25, 29, 33, 37 and 41°C. These temperatures covered the normal thermal range of our study bacterial strain, which had a lower and upper temperature limits of ~ 19 and ~42.2°C, respectively (Lenski & Bennett, 1993; Mongold, Bennett, & Lenski, 1996). Cultures were grown in 4 ml of LB Miller broth (in 50 ml centrifuge tubes), with ~400 rpm shaking. For each assay, the two competitors were first separately grown overnight at 37°C, 1% of which was transferred to fresh medium and grown for 24 hr at each assay temperature for acclimation. Then, the two competitors were added together into a single fresh microcosm (0.02 ml of culture from each), grown in competition for 24 hr at each assay temperature (all cultures could reach a stationary growth phase within the 24 hr of growth, undergoing approximately 6.6 generations, regardless of the assay temperature). The initial and final densities during the course of competition were measured by plating culture dilutions on TA indicator agar plates, where the tested (Ara‐) and the reference (Ara+) strains were distinguished as red and white colonies, respectively. Relative fitness of each tested genotype against the reference strain was estimated from the Malthusian parameters, *W* = *m*_tested_/*m*_reference_, where *m* was calculated as ln (*N*_f_/*N*_0_) with *N*_0_ and *N*_f_ being the relevant initial and final densities, respectively. The fitness of each MA genotype relative to the ancestor was calculated as the difference between the two, analogous to a selection coefficient: *W*_MA_–*W*_ancestor_ (Lopez‐Pascua & Buckling, 2008). Each assay was replicated six times, and the mean value for each assay was used in the subsequent analysis.

### 2.3 Data analyses

We examined several properties of the fitness distributions at every assay temperature, including mean values, standard deviation values, proportion of beneficial mutations (fitness > 0) and proportion of strong‐effect beneficial mutations (fitness > 0.05). Generalized linear models were used for analysing the temperature dependences of those distribution properties, with temperature included as a continuous explanatory variable. Normal errors were used for the analysis of mean and standard deviation values; binomial/quasibinomial errors were used for proportional data, where bound vectors of counts were included as the response variable (e.g. “cbind (count of fitness > 0, 60—(count of fitness > 0))” as the response variable for the analysis of proportion of MA lines with fitness > 0). The “Anova” function provided by the package “car” was used to test for the significance of effects of the explanatory variable in the generalized linear models (*F*‐test was used instead of chi‐square test under conditions of overdispersion). Furthermore, models with both a linear term and a quadratic term of temperature were also performed to test whether there is potential stress across assay environments. Statistical analyses were performed in R 3.5.2.

## 3 RESULTS AND DISCUSSION

### 3.1 Distribution of fitness effects across temperatures

Fitness of a total of 60 MA lines of *E. coli* relative to their ancestral strain was measured across six temperatures. Fitness assays were performed in a rich nutrient medium with aeration, and population sizes in all assay environments were fairly large (>10^8^ cells/ml). In such assay environments, temperature could have directly affected bacterial growth, while its indirect effects through changes in the other environmental factors (such as oxygen availability or the rate of nutrient diffusion) were likely only minimal.

**Figure 2:**
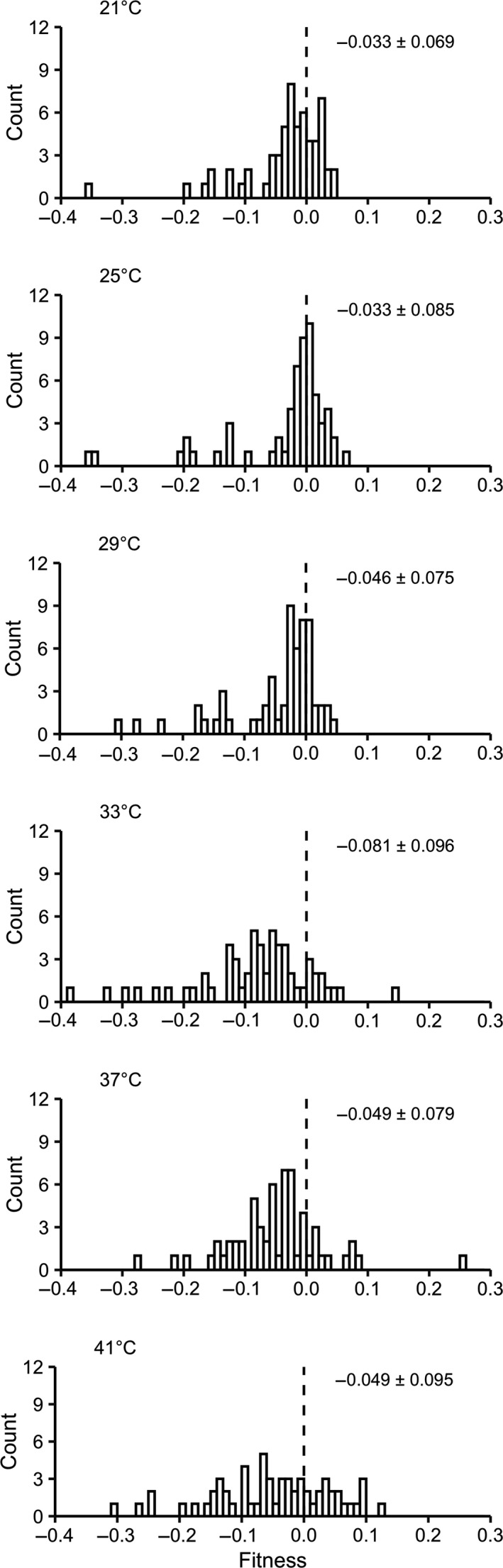
Distributions of fitness values of the 60 MA lines at six assay temperatures. The dashed line in each panel indicates where fitness is zero (equal to the ancestor). Numbers annotated in panels are mean ± *SD* for the total 60 MA lines

**Figure 3:**
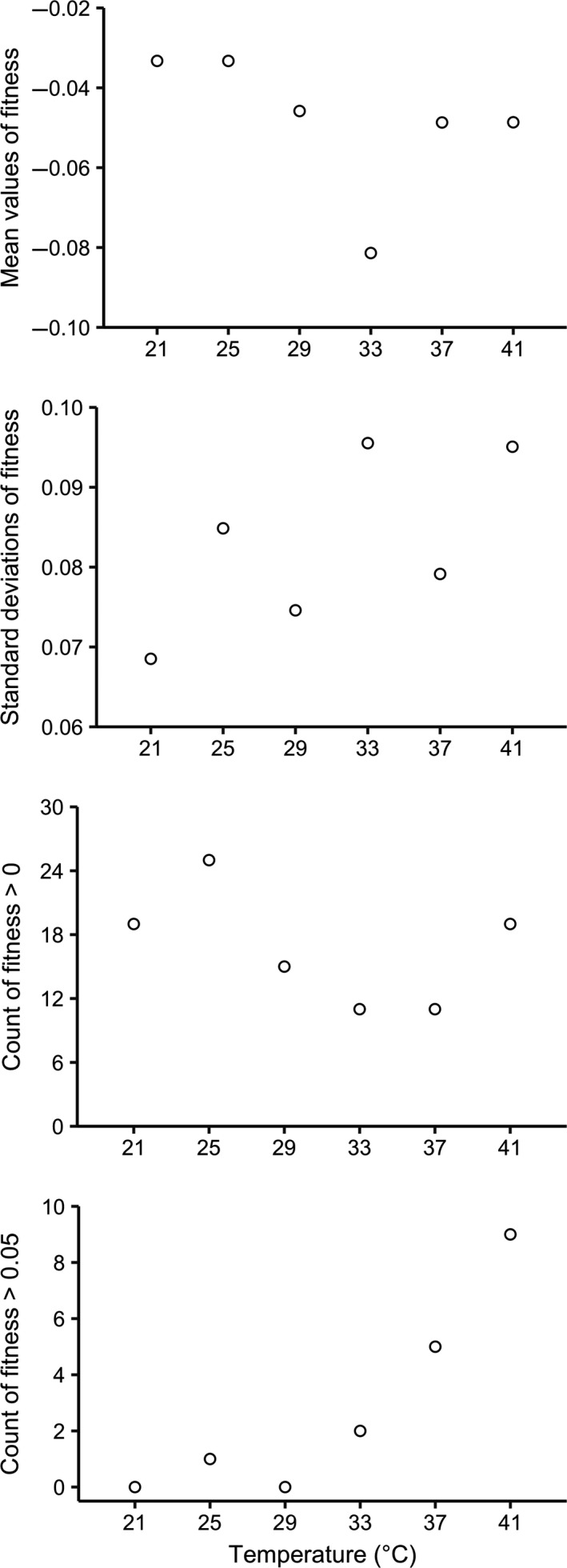
Relationship between fitness distribution properties and assay temperature

Fitness values of the 60 MA lines were overall consistent across the six assay environments (suggested by correlation analysis and variance partitioning analysis; Text S1 and Tables S1 and S2). The observed fitness value distributions were shown in Figure 2. The mean values of the distributions were all smaller than zero (Table S3; one‐sample *t* test, *p* < .002), consistent with the idea that mutations are more likely to be deleterious than beneficial (Bell, 2008; Eyre‐Walker & Keightley, 2007; Lanfear, Kokko, & Eyre‐Walker, 2014; Zeyl & DeVisser, 2001). The mean values did not show a significant relationship with assay temperature (Figure 2 and Figure 3; generalized linear model, $\chi_{1,4}^{2}$ = 1.206, *p* = .272), while the relationship between standard deviation values and temperature was marginally nonsignificant ( $\chi_{1,4}^{2}$ = 3.181, *p* = .075).

Around 1/4 of the MA lines showed fitness advantages against the ancestral strain (fitness > 0), comparable with several earlier short‐term MA experiments (Dickinson, 2008; Trindade et al., 2010). There was not a significant relationship between the proportion of positive fitness values and assay temperature (Figure 2; Figure 3; generalized linear model, *F*_1,4_ = 1.022, *p* = .369). However, the distribution of the positive fitness values did differ among assay environments. Specifically, the proportion of MA lines showing strong fitness advantages (fitness > 0.050) became significantly greater with increasing temperature (Figure 2; Figure 3; $\chi_{1,4}^{2}$ = 21.25, *p* < .001; little change in the result was observed if strong fitness advantages were instead defined as fitness > 0.040 or 0.060, see details in Table S4). Therefore, lower temperatures did not reduce the overall availability of beneficial mutations, but diminished their fitness effects. More detailed analyses based on distribution fitting provided consistent results (Text S1 and Table S5). Meanwhile, we did not find any significant temperature influence on properties of distributions of the negative fitness values, suggesting that the fitness effects of deleterious mutations accumulated in our MA lines are largely insensitive to temperature (Text S1 and Table S6).

It is important to also consider the potential influences of environmental stress on mutational effects for interpreting our results. With an assumption that 37°C is the most benign environment for the *E. coli* strain used in the study, we may have a prediction that the probability of mutations conferring fitness advantages is lowest at 37°C and becomes larger at both lower and higher temperatures. This is because beneficial mutations are expected to be less common in environments to which an organism is already well adapted (or say, near a fitness optimum on the adaptive landscape) (Pal, 1998; Tenaillon, 2014), and mutations may have more variable fitness effects, with a greater chance to confer fitness advantages, when an organism is faced with a less well‐adapted (stressful) environment (Agrawal & Whitlock, 2010; Fisher, 1930; Martin & Lenormand, 2006). The prediction that the 37°C assay environment would see a low probability of beneficial mutations is not supported in the present study. When analysing the relationship between fitness distribution properties and assay temperature, a quadratic term of temperature added into the linear model did not show any significant effects (Table S4). Therefore, it is likely that every temperature we studied here did not cause significant stressful effects on the study organism, though this does not rule out a possible role of environmental stress at more extreme temperatures (which is beyond the scope of the present study).

### 3.2 Caveats with the MA approach for studying mutational effects

The MA strategy has been extensively used for isolating mutations in studies of mutational effects (Dickinson, 2008; Trindade et al., 2010; Zeyl & DeVisser, 2001). However, there are limitations. First, there are typically more than one mutations accumulated in each MA line, and the fitness measured here only reflects the net effects of the multiple mutations, whether additive or epistatic. Second, while this approach minimizes selection, selection against severely deleterious mutations is likely to take place during the MA procedure (Eyre‐Walker & Keightley, 2007; Halligan & Keightley, 2009; Long et al., 2013; Morgan et al., 2014). The operation of negative selection may result in an overrepresentation of beneficial mutations. The occurrence of selection during MA would not be problematic for interpretation of our results as long as the selection is not environment‐specific (as the focus of our study is not a precise description of absolute distribution of fitness effects). We addressed the possibility of environment‐specific selection during MA by examining whether or not MA lines had accumulated mutations that are less deleterious in their “home” environment relative to “foreign” environments. A signal of differential selection was indeed observed for the MA lines of 25°C origin, as the proportion of MA lines with negative fitness values was greater in the “foreign” assay environments relative to the 25°C assay environment (Table S7). Meanwhile, we did not observe such a signal of differential selection for the 28°C (29°C considered as their “home” environment in fitness assays) and 37°C MA lines (Table S7).

Further analysis that excluded the 25°C MA lines did not qualitatively change our results. Specifically, the mean values and standard deviations of the distributions of fitness of the 40 MA lines of 28 and 37°C origin did not show a significant relationship with assay temperature (for mean values, $\chi_{1,4}^{2}$ = 0.059, *p* = .809; for standard deviation, $\chi_{1,4}^{2}$ = 3.325, *p* = .068), nor did the proportion of positive fitness values (*F*_1,4_ = 1.294, *p* = .32). The proportion of MA lines showing strong fitness advantages (fitness > 0.050) became significantly greater with increasing temperature ( $\chi_{1,4}^{2}$ = 23.836, *p* < .001; little change observed if strong fitness advantages were instead defined as fitness > 0.040 or 0.060, $\chi_{1,4}^{2}$ = 20.584, *p* < .026; $\chi_{1,4}^{2}$ = 19.184, *p* < .001, respectively). When a quadratic term of temperature was added into the linear models for the analysis above, its effect was not significant for the mean and *SD* values of the distributions ( $\chi_{1,4}^{2}$ = 2.028, *p* = .154; $\chi_{1,4}^{2}$ = 0.002, *p* = .969, respectively), nor for the proportion of strong beneficial mutations (for proportion of fitness > 0.04, 0.05 and 0.06, $\chi_{1,4}^{2}$ = 0.707, *p* = .401; $\chi_{1,4}^{2}$ = 1.821, *p* = .177; $\chi_{1,4}^{2}$ = 2.724, *p* = .099, respectively).

### 3.3 Implications of our findings

Our observation that higher temperatures allow greater fitness advantages of beneficial mutations provides support for the temperature‐selection speed hypothesis (Fischer, 1960; Rohde, 1992). This hypothesis helps to explain the faster evolution rates and greater magnitude of between‐population divergence in the warmer regions (Fischer, 1960; Gillman, Keeling, Gardner, & Wright, 2010; Gillman, Keeling, Ross, & Wright, 2009; Martin & Mckay, 2004). On the other hand, stronger positive selection may reduce within‐population genetic diversity, contrary to the effect of increased mutation rates. This might be a major reason for the lack of a consistent latitudinal gradient in within‐population genetic diversity (Adams & Hadly, 2012; Hirao et al., 2017; Vellend, 2005; Vellend & Geber, 2005).

Our findings also give implications for understanding contemporary evolution in the face of environmental change, in particular, of pathogenic microbes. For example, the increased crisis of disease transmission with rising temperature has usually been explained by physiological mechanisms such as enhanced parasite reproduction rate (Paaijmans, Read, & Thomas, 2009; Sturrock et al., 2011) and ecological mechanisms including the spread of vector populations (Altizer, Ostfeld, Johnson, Kutz, & Harvell, 2013; Pascual, Dobson, & Bouma, 2009). Our results highlight the possibility that elevation of local temperatures accelerates evolutionary adaptation of pathogens. A recent observational study also reported greater incidence of antibiotic resistance at higher temperatures (MacFadden, McGough, Fisman, Santillana, & Brownstein, 2018); this could, in part, be explained by more rapid adaptation to antibiotic environments, particularly in terms of compensating fitness costs associated with resistance (Bjorkman, Nagaev, Berg, Hughes, & Andersson, 2000).

Cautions should certainly be exercised when extending the results to extremely high temperatures that show stressful effects on organisms. Mutations relevant to thermal stability of proteins may be under stronger negative selection, and protein stability may become a more important determinant of organism growth performance relative to the rates of biophysical and biochemical processes in very hot environments (Chen & Shakhnovich, 2010; Dandage et al., 2018; Echave & Wilke, 2017; Tokuriki & Tawfik, 2009). A recent study combining a biophysical model of protein evolution with empirical data demonstrated that high, stressful, temperatures may generally exacerbate the fitness effects of deleterious mutations and hence suggested that the destabilizing effect of rising temperatures on protein folding would limit the potential for evolutionary adaptation (Berger et al., 2018). More research is clearly needed for the general importance of temperature‐dependent fitness effects of mutations.

## CONFLICTS OF INTEREST

The authors declare no conflict of interest.

## AUTHOR CONTRIBUTIONS

XLC and QGZ designed study; XLC performed experiments; XLC and QGZ analysed data; all authors wrote the paper.

## Supporting information

Supplementary Material

## Data Availability

Data are available at figshare: https://doi.org/10.6084/m9.figshare.7064261.
