# Supplementary material for: Warmer temperatures enhance beneficial mutation effects
Source: J Evol Biol. 2020 Jun 23;33(8):1020–7. doi: 10.1111/jeb.13642 (PMC7496171; doi:10.1111/jeb.13642)
Supplement: Supplementary file 1 — Supplementary Material [file JEB-33-1020-s001.docx]

**Supplementary material**

**Text S1 Supplementary results and discussion**

*Consistency in mutational effects across the assay environments*

We first examined the correlation of fitness of the 60 MA lines between every possible pair of assay environments (using Pearson’s correlation test). We then carried out a variance partitioning analysis. For each possible pair of assay environments, variance in fitness was partitioned into genotypic (${\sigma^{2}}_{G}$), environmental (${\sigma^{2}}_{E}$), and genotype-by-environment interaction (${\sigma^{2}}_{GE}$) components. The ${\sigma^{2}}_{GE}$ component was further decomposed into two parts, responsiveness (*R*) and inconsistency (*I*), as described by Bell (1990; see also Robertson, 1959): ${\sigma^{2}}_{GE}= \sum\frac{\left( \sigma_{Ei}- \sigma_{Ej} \right)^{2}}{2G(G-1)} +\sum\frac{\sigma_{Ei}\sigma_{Ej}\left( 1-\sigma_{EiEj} \right)}{G(G-1)}$, where $\sigma_{Ei}$ and $\sigma_{Ej}$ are the environmental standard deviations of fitness expressed by genotypes *i* and *j*, respectively, and $\sigma_{EiEj}$ is the environmental correlation of fitness across the two genotypes. The responsiveness part reflects the differences in environmental variance among genotypes (genotypes differing in niche breadth). The inconsistency part arises because of the contrasting correlations among genotypes over environments (different genotypes favoring different environments). Then we analyzed how the ${\sigma^{2}}_{GE}$, *R*, and *I* components are correlated with the magnitude of difference between environments (the absolute value of the difference between a pair of assay temperatures), using Mantel tests with 999 permutations in the R package ‘vegan’.

The correlations of fitness values between the assay environments were significantly positive (Table S1), except when extremely different environments were compared (21 versus 37 or 41°C, where non-significant correlations were found). This suggests that the fitness effects of mutations were overall consistent across environments, although becoming less so when environmental dissimilarity became extremely large. The variance partitioning analysis provided results consistent with the correlation analysis. The genotype-by-environment interaction variance (${\sigma^{2}}_{GE}$) was overall lower than the genotypic variance (${\sigma^{2}}_{G}$) by one order of magnitude, suggesting that fitness values of the MA lines were largely consistent across assay environments. Meanwhile, the ${\sigma^{2}}_{GE}$ and its two components, *R* and *I*, showed a positive correlation with the magnitude of the difference between assay environments (Mantel test, *R* = 0.707, *P* = 0.003; *R* = 0.566, *P* = 0.008; *R* = 0.748, *P* = 0.003 for ${\sigma^{2}}_{GE}$, *R* and *I* respectively; see Table S2 for detailed information); this suggests that the mutational effect on fitness became less consistent with increasing environmental dissimilarity.

*Overall effect of assay temperature on fitness of MA lines*

Mixed-effects models (‘lmer’ in the ‘lme4’ package) was used to analyze the effect of MA temperature (evolving temperature) and assay temperature. The model included MA temperature and assay temperature as fixed effects, MA line ID as a random factor, with MA line ID nested within MA temperature which was nested within assay temperature.

Overall, the fitness of the MA lines was not changed by assay temperature and did not differ between the three groups of MA lines (*F*_1,11_ = 2.262, *P* = 0.161; *F*_1,10_ = 2.811, *P* = 0.125; *F*_1,10_ = 1.654, *P* = 0.227, for assay temperature, MA temperature and their interaction respectively).

*Fitting distributions of the positive fitness values*

We investigated the possibility that the observed fitness distributions of beneficial mutations (fitness > 0) in different assay environments could be described by a particular probability distribution. If this is true, the parameters of the fitted distributions may be used for further describing the temperature influences on mutational effects. We considered several distributions that were frequently discussed in the literature of mutational effects: exponential, gamma, Weibull, normal, lognormal and logistic distributions (Shaw *et al.*, 2002; Barrett *et al.*, 2006; Bondel *et al.*, 2019). For the observed positive fitness values in each environment, we used the ‘fitdistr’ function (provide by the package ‘MASS’) to do distribution fitting, obtaining parameter values and Akaike’s Information Criterion (AIC) values. Kolmogorov-Smirnov test was then used to examine the departure of an observed distribution from an expected with parameters specified as those obtained from the ‘fitdistr’ fitting.

The distributions of the positive fitness values across the six assay environments could be described by all the six probability distributions considered here (Table S5). The failure to distinguish between those probability distributions for our data here may result from the relatively small sample size (n ≤ 25; it would be more difficult to reject a null hypothesis that an observed distribution does not differ from an expected one when the sample size is small). Results from fitting the exponential, gamma and Weibull are largely consistent in showing ‘heavier’ right tails in hotter environments. The exponential distribution has a single parameter, and this rate parameter (𝜆) estimated here became non-significantly larger in high-temperature assay environments (linear regression, *F*_1,4_ = 4.663, *P* = 0.097). The fitted gamma distributions were either very exponential-like (when the shape parameter *k* was near 1) or showed ‘heavy’ right tails (in cases being larger than 1, *k* was never larger than 3 and usually smaller than 2); and the estimated rate parameter (*θ*) became smaller at higher temperatures (*F*_1,4_ = 19.57, *P* = 0.011), suggesting ‘heavier’ right tails. Similarly, the fitted Weibull distributions were either exponential-like (when the shape parameter *k* is near one) or had ‘heavy’ right tails (*k* was usually smaller than 2 when larger than 1), and the scale parameter (*𝜆*) was greater for the distributions at high temperatures (*F*_1,4_ = 9.144, *P* = 0.039). The fitted normal distributions suggested greater average mutational effects at higher temperatures (mean value *μ*, *F*_1,4_ = 8.789, *P* = 0.041; standard deviation *σ*, *F*_1,4_ = 4.229, *P* = 0.109). Parameters from the fitted lognormal distribution did not showed significant response to assay temperature (mean value *μ*, *F*_1,4_ = 3.930, *P* = 0.119; standard deviation *σ*, *F*_1,4_ = 1.055, *P* =0.362). The fitted logistic distribution implied ‘heavier’ tails at higher temperatures (location parameter *μ*, *F*_1,4_ = 8.827, *P* = 0.041; scale parameter *s*, *F*_1,4_ = 6.863, *P* = 0.059).

*Distributions of the negative fitness values*

We investigated whether temperature also influenced the fitness effects of deleterious mutations. The proportion of MA lines with negative fitness values did not show a significant relationship with assay temperature (generalized linear model, *F*_1,4_ = 1.022, *P* = 0.369), nor the proportion of the MA lines with strong fitness loss (fitness < -0.05; *F*_1,4_ = 6.16, *P* = 0.068).

Distribution fitting was also performed for the negative fitness values. Absolute values of these negative fitness values were used here as certain probability distributions do not accept negative values. The absolute values of the negative fitness values across the six temperatures could be well described by Gamma, Weibull and lognormal distributions (but not the other three distributions; Table S6). The estimated parameters from fitting of the three distributions suggested little change in the distributions along the temperature gradient (gamma distribution, shape parameter *k*, *F*_1,4_ = 4.856, *P* = 0.092, rate parameter *θ*, *F*_1,4_ = 0.291, *P* = 0.618; Weibull distribution, shape parameter *k*, *F*_1,4_ = 6.247, *P* = 0.067, scale parameter *𝜆*, *F*_1,4_ = 3.38, *P* = 0.140; lognormal distribution, mean value *μ*, *F*_1,4_ = 6.471, *P* = 0.064; standard deviation *σ*, *F*_1,4_ = 3.15, *P* =0.151).

**TABLE S1** Correlations of fitness of the 60 MA lines between assay environments (Pearson’s correlation test, *df* = 58 for each test).

|  | 21°C | 25°C | 29°C | 33°C | 37°C | 41°C |
| --- | --- | --- | --- | --- | --- | --- |
| 21°C |  |  |  |  |  |  |
|  |  |  |  |  |  |  |
| 25°C | *r* = 0.793 |  |  |  |  |  |
|  | *P* = 4.05E-14 |  |  |  |  |  |
| 29°C | *r* = 0.736 | *r* = 0.848 |  |  |  |  |
|  | *P* = 2.18E-11 | *P* < 2.2E-16 |  |  |  |  |
| 33°C | *r* = 0.519 | *r* = 0.710 | *r* = 0.757 |  |  |  |
|  | *P* = 2.15E-5 | *P* = 2.06E-10 | *P* = 2.66E-12 |  |  |  |
| 37°C | *r* = 0.096 | *r* = 0.323 | *r* = 0.406 | *r* = 0.682 |  |  |
|  | *P* = 0.467 | *P* = 0.012 | *P* = 0.001 | *P* = 2.0E-9 |  |  |
| 41°C | *r* = 0.163 | *r* = 0.346 | *r* = 0.375 | *r* = 0.282 | *r* = 0.501 |  |
|  | *P* = 0.213 | *P* = 0.007 | *P* = 0.003 | *P* = 0.029 | *P* = 4.53E-5 |  |

**TABLE S2** Variance components analysis for fitness of the 60 MA lines. Separate analyses were performed for each pair of assay environments. ${\sigma^{2}}_{G}$, ${\sigma^{2}}_{E}$, and ${\sigma^{2}}_{GE}$ refer to variance attributable to genotype (MA line), environment, and G × E interaction. ${\sigma^{2}}_{GE}$ was further decomposed into the responsiveness (*R*) and inconsistency (*I*) components.

|  | 21 °C | 25 °C | 29 °C | 33 °C | 37 °C | 41 °C |
| --- | --- | --- | --- | --- | --- | --- |
| 21 °C |  |  |  |  |  |  |
|  |  |  |  |  |  |  |
|  |  |  |  |  |  |  |
|  |  |  |  |  |  |  |
|  |  |  |  |  |  |  |
| 25 °C | ${\sigma^{2}}_{G}$ =1.056E-02 |  |  |  |  |  |
|  | ${\sigma^{2}}_{E}$ < 1E-7 |  |  |  |  |  |
|  | ${\sigma^{2}}_{GE}$ = 1.334E-03 |  |  |  |  |  |
|  | *R* = 7.859E-04 |  |  |  |  |  |
|  | *I* = 5.485E-04 |  |  |  |  |  |
| 29 °C | ${\sigma^{2}}_{G}$ = 8.888E-03 | ${\sigma^{2}}_{G}$ = 1.175E-02 |  |  |  |  |
|  | ${\sigma^{2}}_{E}$ = 4.728E-03 | ${\sigma^{2}}_{E}$ = 4.723E-03 |  |  |  |  |
|  | ${\sigma^{2}}_{GE}$ = 1.370E-03 | ${\sigma^{2}}_{GE}$ = 1.016E-03 |  |  |  |  |
|  | *R* = 7.196E-04 | *R* = 5.262E-04 |  |  |  |  |
|  | *I* = 6.506E-04 | *I* = 4.901E-04 |  |  |  |  |
| 33 °C | ${\sigma^{2}}_{G}$ = 1.031E-02 | ${\sigma^{2}}_{G}$ = 1.392E-02 | ${\sigma^{2}}_{G}$ = 1.274E-02 |  |  |  |
|  | ${\sigma^{2}}_{E}$ = 6.946E-02 | ${\sigma^{2}}_{E}$ = 6.944E-02 | ${\sigma^{2}}_{E}$= 3.794E-02 |  |  |  |
|  | ${\sigma^{2}}_{GE}$ = 3.513E-03 | ${\sigma^{2}}_{GE}$ = 2.405E-03 | ${\sigma^{2}}_{GE}$ = 1.952E-03 |  |  |  |
|  | *R* = 1.898E-03 | *R* = 1.067E-03 | *R* = 8.978E-04 |  |  |  |
|  | *I* = 1.616E-03 | *I* = 1.338E-03 | *I* = 1.055E-03 |  |  |  |
| 37 °C | ${\sigma^{2}}_{G}$ = 5.998E-03 | ${\sigma^{2}}_{G}$ = 8.902E-03 | ${\sigma^{2}}_{G}$ = 8.310E-03 | ${\sigma^{2}}_{G}$ = 1.285E-02 |  |  |
|  | ${\sigma^{2}}_{E}$ = 7.135E-03 | ${\sigma^{2}}_{E}$ = 7.130E-03 | ${\sigma^{2}}_{E}$= 2.469E-04 | ${\sigma^{2}}_{E}$ = 3.207E-02 |  |  |
|  | ${\sigma^{2}}_{GE}$ = 4.960E-03 | ${\sigma^{2}}_{GE}$ = 4.562E-03 | ${\sigma^{2}}_{GE}$ = 3.517E-03 | ${\sigma^{2}}_{GE}$ = 2.540E-03 |  |  |
|  | *R* = 2.363E-03 | *R* = 2.171E-03 | *R* = 1.631E-03 | *R* = 1.360E-03 |  |  |
|  | *I* = 2.597E-03 | *I* = 2.391E-03 | *I* = 1.886E-03 | *I* = 1.180E-03 |  |  |
| 41 °C | ${\sigma^{2}}_{G}$ = 7.929E-03 | ${\sigma^{2}}_{G}$ = 1.091E-02 | ${\sigma^{2}}_{G}$ = 9.959E-03 | ${\sigma^{2}}_{G}$ = 1.165E-02 | ${\sigma^{2}}_{G}$ = 1.142E-02 |  |
|  | ${\sigma^{2}}_{E}$ = 7.090E-03 | ${\sigma^{2}}_{E}$ = 7.085E-03 | ${\sigma^{2}}_{E}$ = 2.385E-04 | ${\sigma^{2}}_{E}$ = 3.217E-02 | ${\sigma^{2}}_{E}$ = 1.000E-07 |  |
|  | ${\sigma^{2}}_{GE}$ = 5.804E-03 | ${\sigma^{2}}_{GE}$ = 5.329E-03 | ${\sigma^{2}}_{GE}$ = 4.644E-03 | ${\sigma^{2}}_{GE}$ = 6.518E-03 | ${\sigma^{2}}_{GE}$ = 3.880E-03 |  |
|  | *R* = 2.069E-03 | *R* = 1.809E-03 | *R* = 1.623E-03 | *R* = 3.047E-03 | *R* = 1.677E-03 |  |
|  | *I* = 3.735E-03 | *I* = 3.520E-03 | *I* = 3.021E-03 | *I* = 3.471E-03 | *I* = 2.204E-03 |  |

**TABLE S3** Summary of fitness values of the 60 MA lines relative to the ancestor at six temperatures. One-sample *t* test was carried out for the differences of the mean values from zero. Shapiro-Wilk test was used for the departure of the observed distributions from normal. Skewness of the distributions was calculated with Agostino test in the package ‘moments’.

| Temperature (°C) | n | Mean ± SD | t test | Shapiro-Wilk test | Skewness |
| --- | --- | --- | --- | --- | --- |
| 21 | 60 | -0.033 ± 0.069 | *t* = -3.76  *P* = 2×10^-4^ | *W* = 0.802  *P* = 2×10^-7^ | *z* = -5.20  *P* = 2×10^-7^ |
| 25 | 60 | -0.033 ± 0.085 | *t* = -3.04  *P* = 2×10^-3^ | *W* = 0.730  *P* = 4×10^-9^ | *z* = -5.15  *P* = 3×10^-7^ |
| 29 | 60 | -0.046 ± 0.075 | *t* = -4.76  *P* = 7×10^-6^ | *W* = 0.805  *P* = 2×10^-7^ | *z* = -4.47  *P* = 8×10^-6^ |
| 33 | 60 | -0.081 ± 0.096 | *t* = -6.60  *P* = 6×10^-9^ | *W* = 0.937  *P* = 0.004 | *z* = -2.78  *P* = 0.005 |
| 37 | 60 | -0.049 ± 0.079 | *t* = -4.76  *P* = 6×10^-6^ | *W* = 0.948  *P* = 0.012 | *z* = 1.40  *P* = 0.16 |
| 41 | 60 | -0.049 ± 0.095 | *t* = -3.96  *P* = 1×10^-4^ | *W* = 0.974  *P* = 0.221 | *z* = -1.75  *P* = 0.08 |

**TABLE S4** Results of statistical models with only a linear term of the explanatory variable temperature, or both a linear term and a quadratic term.

| Model with linear term | | | | | Model with linear and quadratic terms | | | | |
| --- | --- | --- | --- | --- | --- | --- | --- | --- | --- |
|  | Effect | df | *χ*^2^ or *F* value | P | Effect | df | *χ*^2^ or *F* value | P |  |
| Fitness mean of all MA lines | Linear | 1 | 1.206 (*χ*^2^) | 0.272 | Linear | 1 | 1.724 (*χ*^2^) | 0.189 |  |
|  | Error | 4 |  |  | Quadratic | 1 | 1.463 | 0.226 |  |
|  |  |  |  |  | Error | 3 |  |  |  |
| Fitness SD of all MA lines | Linear | 1 | 3.181 (*χ*^2^) | 0.075 | Linear | 1 | 0.179 (*χ*^2^) | 0.672 |  |
|  | Error | 4 |  |  | Quadratic | 1 | 0.077 | 0.782 |  |
|  |  |  |  |  | Error | 3 |  |  |  |
| Proportion: fitness > 0 | Linear | 1 | 1.022 (*F*) | 0.3692 | Linear | 1 | 1.097 (*F)* | 0.372 |  |
|  | Error | 4 |  |  | Quadratic | 1 | 0.916 | 0.409 |  |
|  |  |  |  |  | Error | 3 |  |  |  |
| Proportion: fitness > 0.040 | Linear | 1 | 10.843 (*χ*^2^) | 0.001 | Linear | 1 | 1.674 (*χ*^2^) | 0.196 |  |
|  | Error | 4 |  |  | Quadratic | 1 | 2.520 | 0.112 |  |
|  |  |  |  |  | Error | 3 |  |  |  |
| Proportion: fitness > 0.050 | Linear | 1 | 21.246 (*χ*^2^) | 4E-06 | Linear | 1 | 0.141 (*χ*^2^) | 0.707 |  |
|  | Error | 4 |  |  | Quadratic | 1 | 0.004 | 0.951 |  |
|  |  |  |  |  | Error | 3 |  |  |  |
| Proportion: fitness > 0.060 | Linear | 1 | 20.895 (*F*) | 0.010 | Linear | 1 | 0.099 (*F*) | 0.774 |  |
|  | Error | 4 |  |  | Quadratic | 1 | 0.002 | 0.968 |  |
|  |  |  |  |  | Error | 3 |  |  |  |

**TABLE S5** Parameter estimates from distribution fitting of the positive fitness values at each assay temperature. Parameters for each distribution are as follows. Gamma: shape parameter *k*, rate parameter *θ*; Weibull: shape parameter *k*, scale parameter *𝜆*; exponential distribution: rate parameter *𝜆*; normal distribution: mean *μ*, standard deviation *σ*; lognormal distribution: mean *μ*, standard deviation *σ*; logistic distribution: location parameter *μ*, scale parameter *s*. D and *P* values were obtained from the K-S test for the departure of the observed distributions from expected ones with parameters specified as those from the model fitting.

| Assay temperature (°C) | n | Gamma | Weibull | Exponential | Normal | Lognormal | Logistic |
| --- | --- | --- | --- | --- | --- | --- | --- |
| 21 | 19 | *k* = 2.912 | *k* = 2.015 | *𝜆* = 43.836 | *μ* = 0.023 | *μ* = -3.962 | *μ* = 0.023 |
|  |  | *θ* = 127.653 | *𝜆* = 0.026 |  | *σ* = 0.012 | *σ* = 0.666 | *s* = 0.007 |
|  |  | AIC = -110.780 | AIC = -112.103 | AIC = -103.658 | AIC = -110.806 | AIC = -108.089 | AIC = -109.858 |
|  |  | D = 0.145 | D = 0.122 | D = 0.272 | D = 0.104 | D = 0.187 | D = 0.102 |
|  |  | *P* = 0.769 | *P* = 0.907 | *P* = 0.098 | *P* = 0.973 | *P* = 0.467 | *P* = 0.977 |
| 25 | 25 | *k* = 1.545 | *k* = 1.284 | *𝜆* = 50.398 | *μ* = 0.020 | *μ* = -4.277 | *μ* = 0.018 |
|  |  | *θ* =77.867 | *𝜆* = 0.022 |  | *σ* = 0.016 | *σ* = 0.903 | *s* = 0.009 |
|  |  | AIC = -144.528 | AIC = -144.275 | AIC = -143.997 | AIC = -132.486 | AIC = -144.025 | AIC = -132.580 |
|  |  | D = 0.105 | D = 0.112 | D = 0.129 | D = 0.176 | D = 0.102 | D = 0.147 |
|  |  | *P* = 0.918 | *P* = 0.880 | *P* = 0.753 | *P* = 0.377 | *P* = 0.935 | *P* = 0.601 |
| 29 | 15 | *k* = 1.077 | *k* = 1.068 | *𝜆* = 65.266 | *μ* = 0.015 | *μ* = -4.710 | *μ* = 0.014 |
|  |  | *θ* = 70.311 | *𝜆* = 0.016 |  | *σ* = 0.013 | *σ* = 1.193 | *s* = 0.008 |
|  |  | AIC = -91.406 | AIC = -91.451 | AIC = -93.354 | AIC = -83.274 | AIC = -89.425 | AIC = -82.820 |
|  |  | D = 0.118 | D = 0.114 | D = 0.122 | D = 0.216 | D = 0.158 | D = 0.184 |
|  |  | *P* = 0.968 | *P* = 0.978 | *P* = 0.959 | *P* = 0.426 | *P* = 0.797 | *P* = 0.624 |
| 33 | 11 | *k* = 1.224 | *k* = 1.051 | *𝜆* = 28.745 | *μ* = 0.035 | *μ* = -3.819 | *μ* = 0.027 |
|  |  | *θ* = 35.174 | *𝜆* = 0.036 |  | *σ* = 0.039 | *σ* = 0.956 | *s* = 0.017 |
|  |  | AIC = -48.152 | AIC = 0.961 | AIC = 0.932 | AIC = -36.395 | AIC = -49.809 | AIC = -39.743 |
|  |  | D = 0.153 | D = 0.141 | D = 0.151 | D = 0.246 | D = 0.171 | D = 0.209 |
|  |  | *P* = 0.925 | *P* = 0.961 | *P* = 0.932 | *P* = 0.447 | *P* = 0.851 | *P* = 0.650 |
| 37 | 11 | *k* = 1.166 | *k* = 1.042 | *𝜆* = 16.237 | *μ* = 0.062 | *μ* = -3.274 | *μ* = 0.050 |
|  |  | *θ* =18.934 | *𝜆* = 0.063 |  | *σ* = 0.066 | *σ* = 1.016 | *s* = 0.030 |
|  |  | AIC = -35.477 | AIC = -35.353 | AIC = -37.320 | AIC = -24.651 | AIC = -36.447 | AIC = -27.321 |
|  |  | D = 0.140 | D = 0.144 | D = 0.143 | D = 0.244 | D = 0.169 | D = 0.189 |
|  |  | *P* = 0.961 | *P* = 0.953 | *P* = 0.956 | *P* = 0.456 | *P* = 0.875 | *P* = 0.759 |
| 41 | 19 | *k* = 1.802 | *k* = 1.564 | *𝜆* = 18.708 | *μ* = 0.053 | *μ* = -3.231 | *μ* = 0.052 |
|  |  | *θ* = 33.719 | *𝜆* = 0.059 |  | *σ* = 0.033 | *σ* = 0.938 | *s* = 0.019 |
|  |  | AIC = -72.614 | AIC = -73.897 | AIC = -71.300 | AIC = -71.966 | AIC = -67.290 | AIC = -70.718 |
|  |  | D = 0.116 | D = 0.100 | D = 0.207 | D = 0.106 | D = 0.173 | D = 0.118 |
|  |  | *P* = 0.933 | *P* = 0.981 | *P* = 0.340 | *P* = 0.968 | *P* = 0.561 | *P* = 0.925 |

**TABLE S6** Parameter estimates from distribution fitting of the negative fitness values at each assay temperature (absolute values used for model fitting). Parameters for each distribution are as follows. Gamma: shape parameter *k*, rate parameter *θ*; Weibull: shape parameter *k*, scale parameter *𝜆*; exponential distribution: rate parameter *𝜆*; normal distribution: mean *μ*, standard deviation *σ*; lognormal distribution: mean *μ*, standard deviation *σ*; logistic distribution: location parameter *μ*, scale parameter *s*. D and *P* values were obtained from the K-S test for the departure of the observed distributions from expected ones with parameters specified as those from the model fitting.

| Assay Temperature (°C) | n | Gamma | Weibull | Exponential | Normal | Lognormal | Logistic |
| --- | --- | --- | --- | --- | --- | --- | --- |
| 21 | 41 | *k* = 0.980 | *k* = 0.956 | *𝜆* = 16.880 | *μ* = 0.059 | *μ* = -3.416 | *μ* = 0.047 |
|  |  | *θ* = 16.542 | *𝜆* = 0.058 |  | *σ* = 0.068 | *σ* = 1.195 | *s* = 0.032 |
|  |  | AIC = -145.753 | AIC = -145.892 | AIC = -147.742 | AIC = -100.665 | AIC = -145.174 | AIC = -110.633 |
|  |  | D = 0.105 | D = 0.097 | D = 0.107 | D = 0.232 | D = 0.094 | D = 0.194 |
|  |  | *P* = 0.718 | *P* = 0.801 | *P* = 0.698 | *P* = 0.020 | *P* = 0.833 | *P* = 0.080 |
| 25 | 35 | *k* = 0.605 | *k* = 0.709 | *𝜆* = 14.045 | *μ* = 0.071 | *μ* = -3.662 | *μ* = 0.054 |
|  |  | *θ* = 8.500 | *𝜆* = 0.057 |  | *σ* = 0.092 | *σ* = 1.652 | *s* = 0.047 |
|  |  | AIC = -118.183 | AIC = -118.944 | AIC = -112.961 | AIC = -63.513 | AIC = -117.875 | AIC = -67.484 |
|  |  | D = 0.136 | D = 0.111 | D = 0.243 | D = 0.269 | D = 0.111 | D = 0.244 |
|  |  | *P* = 0.498 | *P* = 0.741 | *P* = 0.027 | *P* = 0.010 | *P* = 0.737 | *P* = 0.026 |
| 29 | 45 | *k* = 0.883 | *k* = 0.898 | *𝜆* = 15.108 | *μ* = 0.066 | *μ* = -3.379 | *μ* = 0.052 |
|  |  | *θ* = 13.333 | *𝜆* = 0.063 |  | *σ* = 0.075 | *σ* = 1.236 | *s* = 0.038 |
|  |  | AIC = -150.851 | AIC = -151.301 | AIC = -152.371 | AIC = -101.817 | AIC = -153.358 | AIC = -106.368 |
|  |  | D = 0.144 | D = 0.129 | D = 0.167 | D = 0.229 | D = 0.091 | D = 0.214 |
|  |  | *P* = 0.283 | *P* = 0.406 | *P* = 0.144 | *P* = 0.015 | *P* = 0.814 | *P* = 0.028 |
| 33 | 49 | *k* = 1.728 | *k* = 1.358 | *𝜆* = 9.306 | *μ* = 0.107 | *μ* = -2.547 | *μ* = 0.094 |
|  |  | *θ* = 6.085 | *𝜆* = 0.118 |  | *σ* = 0.083 | *σ* = 0.917 | *s* = 0.043 |
|  |  | AIC = -124.105 | AIC = -123.456 | AIC = -118.608 | AIC = -100.460 | AIC = -115.064 | AIC = -105.670 |
|  |  | D = 0.087 | D = 0.101 | D = 0.172 | D = 0.178 | D = 0.106 | D = 0.118 |
|  |  | *P* = 0.818 | *P* = 0.665 | *P* = 0.096 | *P* = 0.080 | *P* = 0.599 | *P* = 0.468 |
| 37 | 49 | *k* = 1.707 | *k* = 1.350 | *𝜆* = 13.617 | *μ* = 0.073 | *μ* = -2.932 | *μ* = 0.066 |
|  |  | *θ* = 23.245 | *𝜆* = 0.080 |  | *σ* = 0.057 | *σ* = 0.862 | *s* = 0.030 |
|  |  | AIC = -161.109 | AIC = -160.369 | AIC = -155.913 | AIC = -138.004 | AIC = -158.856 | AIC = -141.391 |
|  |  | D = 0.072 | D = 0.073 | D = 0.149 | D = 0.134 | D = 0.093 | D = 0.124 |
|  |  | *P* = 0.948 | *P* = 0.937 | *P* = 0.207 | *P* = 0.315 | *P* = 0.754 | *P* = 0.404 |
| 41 | 41 | *k* = 1.447 | *k* = 1.280 | *𝜆* = 10.423 | *μ* = 0.096 | *μ* = -2.728 | *μ* = 0.087 |
|  |  | *θ* = 15.087 | *𝜆* = 0.103 |  | *σ* = 0.074 | *σ* = 1.016 | *s* = 0.041 |
|  |  | AIC = -109.292 | AIC = -109.759 | AIC = -108.212 | AIC = -93.403 | AIC = -101.999 | AIC = -94.380 |
|  |  | D = 0.059 | D = 0.057 | D = 0.122 | D = 0.150 | D = 0.118 | D = 0.117 |
|  |  | *P* = 0.997 | *P* = 0.998 | *P* = 0.537 | *P* = 0.289 | *P* = 0.578 | *P* = 0.593 |

**TABLE S7** Frequency of negative fitness values for the three groups of MA lines across the six assay environments. Analysis was performed based on the frequency data for the possibility that MA lines had accumulated mutations that are less deleterious in their ‘home’ environment relative to ‘foreign’ environments. This was done by examining whether the proportion of negative fitness values for a group of MA lines was greater in alternate environments relative to the ‘home’ environment, using the ‘prop.test’ function. The 29°C assay environment was considered as ‘home’ environment for MA lines of 28°C origin. For the 25°C MA lines, the proportion of negative fitness values in the 29, 33, 37, and 41°C assay environments were lower than that at 25°C (*P* < 0.05). No such signal of environment-specific selection was found for the 28 and 37°C MA lines (*P* > 0.20).

| Assay temperature (°C) | MA groups | | |
| --- | --- | --- | --- |
|  | The 25°C MA lines | The 28°C MA lines | The 37°C MA lines |
| 21 | 9/20 | 15/20 | 17/20 |
| 25 | 8/20 | 16/20 | 11/20 |
| 29 | 16/20 | 12/20 | 17/20 |
| 33 | 17/20 | 15/20 | 17/20 |
| 37 | 18/20 | 15/20 | 16/20 |
| 41 | 19/20 | 9/20 | 13/20 |

SUPPLEMENTARY REFERENCES

Barrett, R.D.., Craig MacLean, R. & Bell, G. 2006. Mutations of intermediate effect are responsible for adaptation in evolving *Pseudomonas fluorescens* populations. *Biol. Lett.* **2**: 236–238. http://doi: ﻿10.1098/rsbl.2006.0439

Bondel, K.B., Kraemer, S.A., Samuels, T., McClean, D., Lachapelle, J., Ness, R.W., *et al.* 2019. Inferring the distribution of fitness effects of spontaneous mutations in *Chlamydomonas reinhardtii*. *PLoS Biol.* **17**: e3000192. http://doi: ﻿ort/10.1371/journal.pbio.3000192

Shaw, F.H., Geyer, C.J. & Shaw, R.G. 2002. A comprehensive model of mutations affecting fitness and inferences for *Arabidopsis thaliana*. *Evolution.* **56**: 453–463. http://doi: ﻿10.1111/j.0014-3820.2002.tb01358.x
